# Seen but overlooked: animals as stakeholders in AI ethics discourses

**DOI:** 10.1093/af/vfaf057

**Published:** 2026-01-09

**Authors:** Mona F Giersberg

**Affiliations:** Department of Population Health Sciences, Faculty of Veterinary Medicine, Animals in Science and Society, Utrecht University, Utrecht, 3584 CM, The Netherlands

**Keywords:** artificial intelligence, collaboration, perspective, responsibility, values

Implications

- Ethics of artificial intelligence is a field of applied ethics that is concerned with novel and applied normative questions that arise with the development and use of artificial intelligence. In practice, it is important to engage with the ethics of artificial intelligence to understand these technologies in a societal context, and to be able to ­anticipate and act upon concerns they may raise.
- Animals as individuals with moral relevance are largely ignored in current considerations of ethics of artificial intelligence. While animal ethicists increasingly see ­animals as stakeholders in this context, they are still overlooked in practical discourses about the responsible development and use of artificial intelligence.
- We need to represent animal perspectives and interests explicitly to move toward a “responsible development and use” of artificial intelligence which is also responsible for animals.
- To address this, we need to integrate knowledge from different fields of applied ethics, agricultural and biological sciences, and practical experiences.

## Introduction

Artificial intelligence (AI) is increasingly influencing our daily lives. This certainly also applies to contexts in which we breed, keep or otherwise interact with animals. Precision Livestock Farming, a method of animal agriculture, draws on the necessary components of AI: data, computing, and modelling. With the concepts and technologies of process engineering, multimodal data is increasingly being processed and analyzed by means of AI (Tuyttens et al., 2022). In veterinary medicine, AI-based tools are used, for instance, in diagnostic imaging (Boissady et al., 2020) or to detect pain in cats (Steagall et al., 2023). In domestic ­settings, commercially available wearable sensors for dogs promise to provide health information to their keepers, such as sleep scores and activity indices (Hilborn et al., 2024). In addition, AI-based technologies are developed and applied across the field of animal sciences to study various animal behaviors and conditions (e.g., Chen et al., 2020; Volkmann et al., 2021; Van Putten et al., 2025).

It is often assumed or even claimed that such technologies would only benefit the animals subjected to them. These benefits may be direct (e.g., a smart ventilation system that automatically adjusts the ambient conditions in a barn based on outdoor weather conditions) or indirect through more information that would lead to improved management or care (e.g., an activity tracker that alerts the caregiver of a dog when the dog’s activity patterns deviate from the daily average). However, it is also common knowledge that the development and use of AI in general is associated with several ethical challenges, such as transparency, responsibility and (or) discrimination. Risks of AI for animals do not only include direct threats to their welfare, but also harms that arise from how we as humans perceive animals and interact with them (Tuyttens et al., 2022; Coghlan and Parker, 2023). The potential of AI to mediate, enhance, disrupt or simply change human-animal relationships asks for analysis of and systematic reflection on the normative questions that may arise against this background. Being able to approach this requires familiarizing oneself with the field of ethics of AI and understanding why it is important to include animals in this context.

In this article, I use a broad definition of AI as technical detail is not necessary to explore the field of ethics of AI, and fixed or narrow definitions may quickly become outdated. Here, I regard AI as technologies with varying levels of autonomy and the capacity to process data in ways, such as classifying, predicting and inferring, which resemble intelligent behavior (UNESCO, 2022). However, I restrict my elaborations to AI-based technologies which are intentionally deployed to manage animals or information about them. Other technologies, such as self-driving cars that can accidentally harm animals (Black and Fenton, 2021), are beyond the scope of this article. To develop and use AI systems, the following key components are needed: data (high quality, relevant and in large amounts), algorithms (i.e., a set of rules that AI systems follow to process data and make decisions, includes various models), and compute resources. I build my arguments on the assumption that these components are of high quality and function technically correctly. Threats to animals that arise due to low data quality or system failure are discussed elsewhere (e.g., Tuyttens et al., 2022). I use the term “animals” to distinguish between humans and nonhuman animals. In particular, I focus on sentient animals (Browning and Birch, 2022) kept by humans for various ends (e.g., food production, companionship, research, education, conservation), though some aspects may also be relevant to applying AI to process information about wildlife.

In this article, I first introduce ethics of AI as a field of research and its practical implications and importance. This introduction to the field is by no means exhaustive. It is particularly aimed at providing an overview for researchers and professionals whose interest in ethics and AI stems from other disciplines, such as agricultural and biological sciences. Second, I address the point that animals as morally relevant individuals are currently being ignored in practical discourses about the responsible development and use of AI, and argue why this is problematic. Third, I propose future directions on how to integrate animal perspectives into such discourses. To facilitate ethical reflection, I close by inviting researchers and professionals to ask themselves a number of questions when developing AI-based technologies for and applying them to animals.

## What Is Ethics of AI and Why Is It Important?

The current pace at which AI-based technologies are developed and introduced to the market is outpacing a general, basic understanding of how these technologies work and what the actual aims of their use are. At the same time, AI will inevitably have a significant impact on many areas of our lives. As a typical response to novel technologies, fundamental questions are asked, which often focus on concerns (Müller, 2025). These include questions about what we should do with AI, what AI should do, what risks AI entails, and how we can manage these risks. Analyzing and reflecting on questions like these in a systematic way is the subject of the field of ethics of AI.

**Figure 1.**
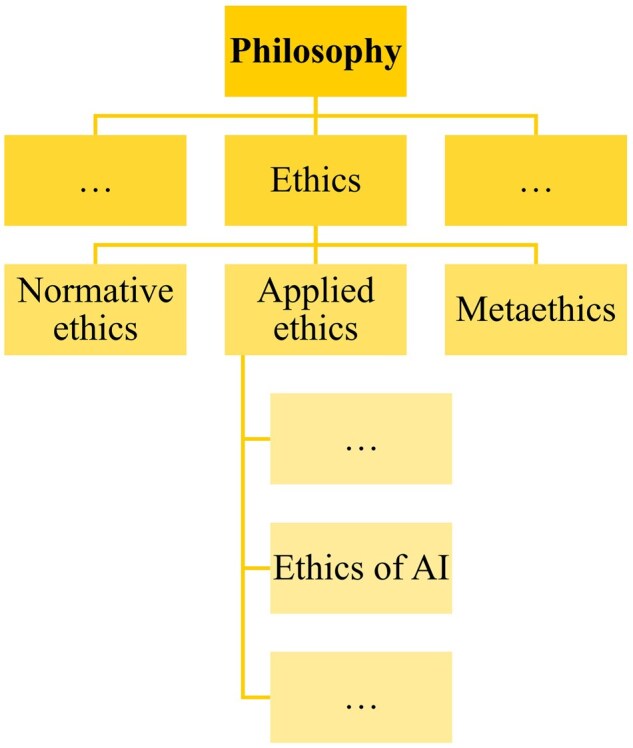
Positioning the field of ethics of AI within the discipline of philosophy and the branch of ethics. “…”stands for other branches and fields not addressed in this article.

Ethics is a major branch of philosophy that studies moral principles or questions of what people ought to do (Figure 1). Within ethics, we distinguish between three branches: normative ethics, which seeks to define general principles to guide human action; metaethics, which investigates the fundamental assumptions and concepts of ethics; and applied ethics, which addresses concrete ethical issues and also covers ethics of AI.

As ethics of AI is a dynamic and relatively new field of applied ethics, it is challenging to provide a concise overview (Müller, 2025) or to define which issues actually fall under ethics of AI and which do not (Lambrecht and Moreno, 2024). A broad distinction can be made between normative questions that are raised in connection with AI as object or tool for human use (e.g., translation programs), with AI as subject or moral agent with certain responsibilities and rights (e.g. human-like robots), and those questions that arise with “singularity,” i.e., a certain point at which AI exceeds levels of human intelligence and becomes out of human control and prediction (Müller, 2025). In the context of animal sciences, we currently deal mainly with issues related to the use of AI-based technologies as objects (e.g., behavior tracking algorithms).

Due to the tremendous interest in AI, the academic field of ethics of AI faces a number of challenges. These include, for instance, dilution, when researchers add an AI aspect that is only marginally relevant to their actual research question or conceptual bloating, when researchers develop new terms and concepts to investigate ethical issues related to AI that could equally be investigated by using established terms and concepts (Lambrecht and Moreno, 2024). To prevent dismission of the field as “all hype and no substance,” Lambrecht and Moreno (2024) introduced a definition of and a methodological framework for ethics of AI. According to the authors, a question needs to be either novel, i.e., new concepts are necessary to explain a phenomenon of AI-based technologies, or applied, i.e., gained new importance in the sense of greater urgency or greater stakes because of AI, to count as question of AI ethics (Lambrecht and Moreno, 2024). In animal sciences, such applied questions of ethics of AI may be related to concerns about animal welfare. Although ­ethical issues regarding animal welfare are well understood from a conceptual perspective (e.g., Tannenbaum, 1991), the introduction of AI-based technologies may lead to different or more severe threats to animal welfare (Tuyttens et al., 2022).

However, we need to engage with ethics of AI also apart from mere academic interest. That is because these new technologies with their potential to significantly impact people’s lives, require us as a society to form an answer as to how we want them to be developed and used. This societal answer can be expressed in the form of guidelines, regulations and laws with the final aim to achieve a “responsible development and use” (Müller, 2025) of AI-based technologies. Ideally, new insights from the field of ethics of AI would inform such a societal answer. However, in practice, this process seems to be less straightforward. Over the past years, numerous guidelines, recommendations and frameworks on “ethical AI” have been published by various public and private organizations which, due to their loose combination of abstract principles, are an invitation to engage in several malpractices (Floridi, 2019). These include, for instance, “ethics shopping” which means that organizations do not choose guidelines or principles that require them to change their practices in order to comply with ethical standards, but rather those that can serve as a retrospective justification for already established practices (Floridi, 2019).

**Figure 2.**
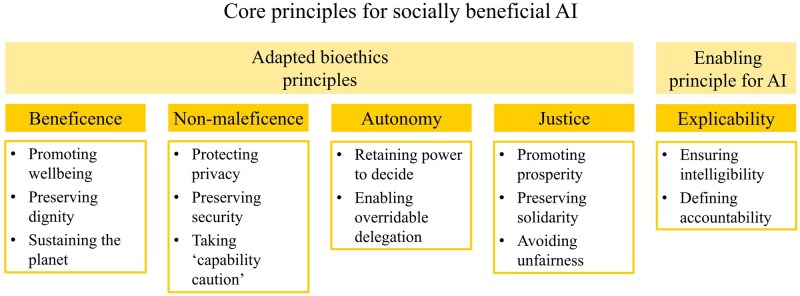
Core principles for socially beneficial AI as identified by Floridi and Cowls (2019). Four of them correspond to the core principles of bioethics and one is an enabling principle.

In their review of high-profile recommendations from reputable, multi-stakeholder organizations, Floridi and Cowls (2019) identified 47 principles aiming at an ethical development and use of AI. However, as these principles show a high degree of overlap, the authors condensed them into five core principles ­(Figure 2). Four of them correspond to the core principles of bioethics, which are: beneficence, nonmaleficence, autonomy and justice (Beauchamp and Childress, 1979; Floridi and Cowls, 2019). According to the authors, beneficial AI supports human wellbeing, preserves human dignity, and contributes more broadly to environmental sustainability. Adhering to the principles of privacy, security, and capability caution, i.e., assuming that there are no limits in what AI may achieve and planning accordingly, is classified as nonmaleficence. Autonomy basically means that humans retain the power to decide and that delegations of decision-making to an AI-based technology are always reversible. The principle of justice points to shared prosperity from AI, preserved solidarity, and avoiding unfair AI. Floridi and Cowls (2019) introduce a fifth, enabling principle for socially beneficial AI: explicability. Explicability covers intelligibility, i.e., AI-based technologies need to be understandable and ­interpretable in a technical sense so that people know which benefits or harms they may bring about, and accountability, i.e., people need to know who can be held responsible for how the technology is working (Floridi and Cowls, 2019).

From the academic field of ethics, Floridi and Cowls’ (2019) principles may be subject to similar criticism as the original bioethics principles. At that time, criticism emphasized that the bioethics principles are not principles in the traditional sense which summarize an elaborate ethical theory, but that they borrow from various theories, therefore lack systematic relationship, and fail to guide concrete action (Clouser and Gert, 1990). In practice, the principles for socially beneficial AI may be criticized for being too abstract or too loosely defined to be effective (Lambrecht and Moreno, 2024). They therefore need to be seen as a framework within which more concrete guidelines or rules can be formulated for specific sectors, such as the animal sciences sector (Floridi and Cowls, 2019). In this sense, the principles may serve as a minimum common ground in contexts across disciplines and cultures in order to form a societal answer and get one step closer toward responsible development and use of AI.

In summary, ethics of AI is a field of applied ethics that is concerned with novel and applied normative questions that arise with the development and use of AI. In practice, it is important to engage with ethics of AI to understand these technologies in a societal context, and to be able to anticipate and act upon concerns they may raise. A condensed set of principles for socially beneficial AI (Floridi and Cowls, 2019) can aid this process, also in disciplines that might be less familiar with ethical theories, such as animal sciences.

## Animals as Overlooked Stakeholders

Until recently, the academic field of ethics of AI seemed to have exclusively focused on the risks and benefits of AI-based technologies for humans or humanity as a whole. Currently, a group of researchers, often from other fields of applied ethics, is advocating for the inclusion of animals in AI ethics ­considerations (e.g., Bossert and Hagendorff, 2021; Coghlan and Parker, 2023; Singer and Tse, 2023). In summary, these authors and a growing number of other animal ethicists point out that although animals are directly (e.g., by sensor-controlled feeding stations) and indirectly (e.g., through alterations in human–animal relationships), intentionally (e.g., by tracking certain animals) and unintentionally (e.g., by harming them by self-driving cars) impacted by AI in various contexts, they are not, or only collectively (as part of ‘nature’), considered in deliberations of ethics of AI. This is seen as problematic because animals are individuals with interests (e.g., to avoid pain and experience pleasure) and we as humans have reason to consider them morally. Although people disagree about the moral status of animals, there seems to be a consensus that animal interests need to be considered at least to some extent (Janssens, 2022). The fact that animal interests do count to some extent is demonstrated in practice, for instance, by animal welfare laws that exist in many countries for many contexts in which humans keep or interact with animals. If then animal interests are at stake due to the development and use of AI-based technologies and we as humans take animal interests seriously in some way, we need to include ­animals in our academic and practical considerations of ­ethics of AI.

These ideas have not yet reached other academic fields. Overview articles on ethics of AI (e.g., Müller, 2025) continue to take no notice of animals. However, what is more striking is that animals are also hardly considered in practical settings, i.e., where actual AI-based technologies are developed and used for or on actual animals by actual researchers, farmers, veterinarians or other professionals. When ethical aspects, such as fairness, trust, agency, privacy and security are bought up, for instance, by stakeholders of the animal agriculture sector, they refer to the humans using AI-based or other digital technologies and not to the animals subjected to them (Schillings et al., 2023). Similar talk can be observed among animal scientists, where concerns regarding the development and use of certain AI-based technologies are usually related to humans. Although animals may be seen as central stakeholders in research projects ­(Giersberg et al., 2024), the use of AI is often judged as merely positive for the animals. This judgement is particularly prevalent in the case of technologies that contain only noninvasive elements (e.g., cameras or sound recorders as hardware components). People often claim that the animals would be unaware of the data collection process, and after all, the technology would be introduced to improve animal welfare. While there is far more to say about this argumentation, I need to limit myself here to pointing out that it ignores those values and meanings humans attribute to a “good life” for animals that go beyond how animals perceive their own situation (cf. Bovenkerk, 2020).

**Figure 3.**
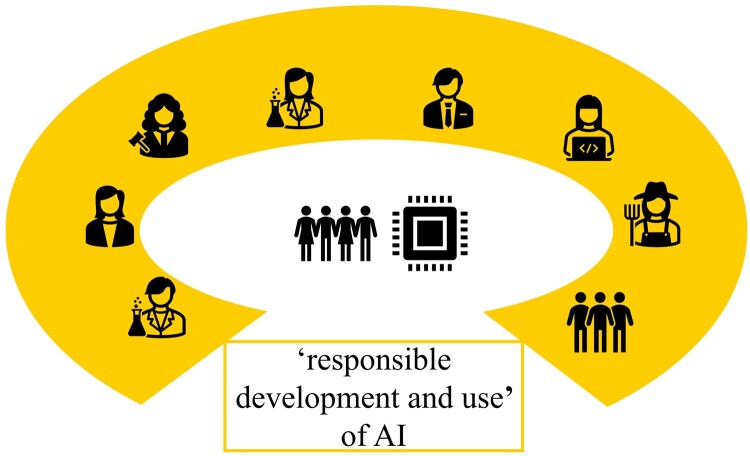
Multistakeholder consortia engage in practical discourses about the “responsible development and use” of AI with the aim to benefit humans or humanity as a whole.

Returning to the set of principles for socially beneficial AI, it is clear that, for instance, preserving “dignity” and “privacy” to account for beneficence and nonmaleficence means preserving “human dignity” and “human privacy.” From this perspective, also concrete guidelines for specific sectors or cases will be formulated by relevant multi-stakeholder consortia in practical discourses (Figure 3). Overlooking animals in these discourses, particularly in contexts where AI-based technologies are developed and used for or on animals, contrasts with the reality of other fields of research, in which the concepts the principles refer to are naturally studied with regard to animals. One example is the concept of dignity, a concept whose extension to animals has been the subject of vivid discussions in applied ethics (e.g., ­Nussbaum, 2008:254–255). Another example is the concept of privacy, for which a biological basis has been found in humans and animals, and which therefore deserves more species-inclusive consideration (e.g., Paci et al., 2022).

It is beyond the scope of this article to discuss whether, to which extent or how concepts like dignity and privacy, which play an important role in the principles for socially beneficial AI for humans, can or should be extended to animals. My point is that we need to undertake this inquiry and face these ­questions, not only in a theoretical context and not only within the field of applied ethics, but in practice, in disciplines such as animal sciences, where actual AI-based technologies are developed and used on or around actual animals. Ignoring animals in AI ethics discourses is not an option. Obviously, this does not mean that we should not develop or use AI-based technologies for or on animals at all. It may even be the best option to use AI in a given situation. Similarly, ethics of AI is not about prohibiting all uses of AI in all human contexts either, it tries to solve issues for which it is not readily known what the right thing to do is. In the considerations and discourses associated with this, animals must be recognized as stakeholders and taken seriously.

Why does this claim seem so difficult to realize in practice so far? First, the development and use of AI-based technologies in animal sciences mostly takes place in transdisciplinary projects or contexts. The stakeholders involved often come from different epistemic backgrounds, which makes mutual understanding and effective collaboration challenging in general (Giersberg et al., 2022). Second, animals, who lack self-reporting capacities and are in a vulnerable, dependent position, add an additional complexity to the situation which may be convenient to ignore (Giersberg and Meijboom, 2022). This problem is not new and not unique to the case of AI. Animals as stakeholders have been overlooked in plain sight in other contexts, for instance, in considerations of sustainability (Vinnari and Vinnari, 2022).

In summary, animals as individuals with moral relevance are largely ignored in current considerations of ethics of AI. While animal ethicists increasingly see animals as stakeholders in this context, they are still overlooked in practical discourses about the responsible development and use of AI. This is problematic because we as humans have agreed that animal interests matter and have to be taken into account at least to some extent.

## How to Proceed?

Aligning the field of ethics of AI with animal ethics appears to be an academic exercise which has already been set in motion by, for instance, the work of Singer and Tse (2023). The far greater challenge lies in the practical inclusion of animal perspectives, which comes with a certain urgency given the pace at which AI-based technologies are developed and used for and on animals. As with the development of AI-based technologies in animal sciences in general, a first step is, despite the challenges outlined above, to implement a transdisciplinary approach. To be successful, such an approach needs to facilitate an interaction of conceptual consideration and practical efforts (Giersberg et al., 2022). It needs to create moments for genuine reflection from the start of developing or planning to use AI-based technologies in an animal context. These moments can be organized as workshops or practical discourses to help participants to be aware of, to acknowledge and to contribute to the plurality of stakeholders, their interests and their views on the topic (Giersberg et al., 2024). Having an idea of what ethics of AI is about, as outlined above, can help stakeholders from the agricultural and biological sciences to engage more constructively with stakeholders from the field of ethics of AI.

**Figure 4.**
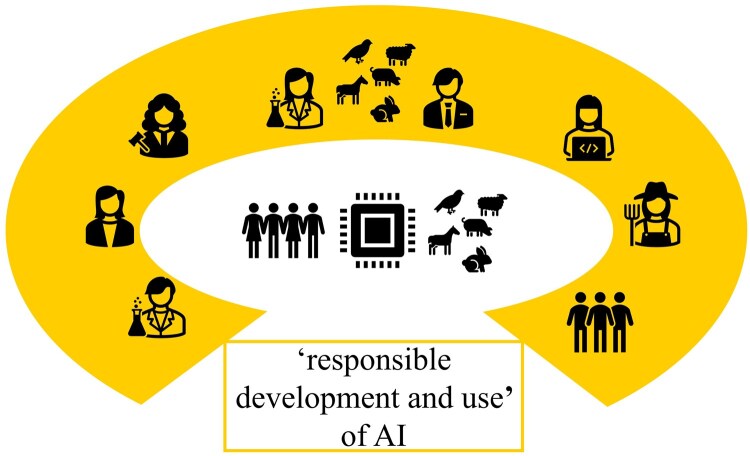
Including animals in practical discourses about the “responsible development and use” of AI with the aim to benefit humans and animals.

**Figure 5.**
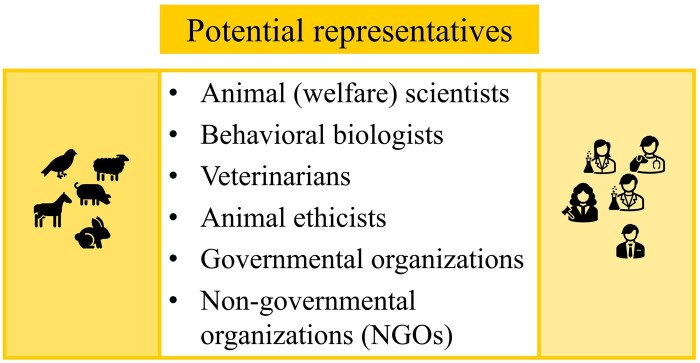
Potential candidates who can represent the animal’s perspectives and interests in practical discourses.

This approach should not be confused with discourse ethics as coined by Habermas and Apel, whose ideal is a free, rational argumentative community, and whose principles exclude paternalism and do not consider an inclusion of the interests of stakeholders incapable of argumentation. If we are to include animals in practical discourses about responsible development and use of AI, we cannot avoid the need for representatives who are capable of arguing the animals’ case (Figure 4). In certain cases, we may be able to expose animals to a technology (e.g., sensor-controlled feeding) and take their own responses to it into account in the argumentation. However, even in such cases, we rely on other stakeholders (e.g., behavioral biologists) who can interpret the responses of the animals and on those (e.g., animal ethicists) who can analyze potential impacts of such technology use on our broader relationship with animals (e.g., in terms of instrumentalization or objectification of animals, cf. Bovenkerk, 2020). Potential candidates who can represent the perspective and interests of the animal in practical discourses are animal (welfare) scientists, behavioral biologists, veterinarians, animal ethicists, and governmental organizations and nongovernmental organizations specialized in animal topics (Figure 5). Ideally, they work together to represent the animal from as many angles as possible. Obviously, these stakeholders need to draw on their scientific and experiential expertise, which comes with a level of uncertainty, as it will never be possible for humans (or human-created AI) to represent the animal in a way that does full justice to its unique “animalness.”

It is important that facilitation efforts, which are essential in all transdisciplinary collaborations (Giersberg et al., 2024), ensure that the animal’s case is taken seriously and is given equal consideration as the other stakeholders. Otherwise, there is the risk that the inclusion of animals in practical discourses about the development and use of AI-based technologies will become a tick-box exercise that is easily overridden by other stakeholder interests. In practice, it may also help to have a physical item representing the animal at the table.

By having animal perspectives and interests explicitly represented when developing and using AI in animal sciences, we can move closer toward more animal-centered AI-based technologies. This does not mean that we should fall for the same problematic assumptions as when talking about human-centered AI (Ryan, 2025). What it means is that we need to give our best to ensure that “responsible development and use of AI” does also mean “responsible” for the animals.

## Conclusions and Invitations

In this article, I provided a brief introduction to the field of ethics of AI including a condensed set of principles for socially beneficial AI that may be useful for practical considerations in animal sciences. I further addressed the point that animals, who are currently largely overlooked in such considerations, need to be included in practical discourses about the responsible development and use of AI. I proposed the implementation of a transdisciplinary approach in which animals are represented and taken seriously. I close by posting a set of questions which can support researchers and professionals to take a first step in this process of explicit animal inclusion.

The next time practical discourses about the responsible development and use of AI-based technologies take place, for instance, in the context of an animal science research project or the implementation of a new AI tool in veterinary practice, researchers and professionals may ask themselves the following questions:

Which of the principles for socially beneficial AI (Figure 2) underlay the technology in question?

Do these include animals? If so, how?

Is it relevant to include animals in these considerations? Why/why not?

What would need to change in order to include animals?

Who benefits from the development and/or use of the technology?

Do animals benefit from it? If so, how? If not, is it relevant?

What would need to change for animals to benefit from the technology?

In answering and discussing these questions, one should not be limited by the assumption that something is not feasible within current practices or within current structures of a particular sector. Nor should one restrict their arguments to aspects of animal welfare only or draw the conclusion that more information automatically means improved animal management or care. In addition, it is useful to consider the development and use of AI-based technologies for or on animals in the broader context in which humans keep animals. Does the technology genuinely help to improve animal lives or is it just a technological fix to an inherently detrimental practice? If we integrate the knowledge from different fields of applied ethics, agricultural and biological sciences, and practical experiences, we have a chance to tackle these issues. We should take this chance and collaborate to make the current ‘AI summer’ more species-inclusive.

## About the Author

Mona F. Giersberg is Associate Professor and coordinator of the AI & Animal Welfare Lab at Utrecht University, the Netherlands. Her work is located at the crossroads of (animal) welfare, sensor- and AI-based technologies, and ethics. As a researcher she sought collaboration with working groups in the fields of humanities and social sciences and expanded her methodologies to include various empirical and nonempirical approaches from these disciplines. Currently, she works on a systematic and strategic approach to transdisciplinary collaboration in the area of responsible AI for animal welfare. Giersberg holds a PhD on the topic of biometric data of farm animals and computer-based techniques to collect these. It was defended with honors and received several awards. Before moving to Utrecht, Giersberg worked as a researcher at Wageningen University and Research (NL) and at the University of Veterinary Medicine Hannover, Foundation (DE).
